# Role of Tau Protein in Neurodegenerative Diseases and Development of Its Targeted Drugs: A Literature Review

**DOI:** 10.3390/molecules29122812

**Published:** 2024-06-13

**Authors:** Jiakai Yang, Weijia Zhi, Lifeng Wang

**Affiliations:** 1 Graduate Collaborative Training Base of Academy of Military Medical Sciences, Hengyang Medical School, University of South China, Hengyang 421001, China; yjk815ue@163.com; 2 Beijing Institute of Radiation Medicine, 27 Taiping Road, Beijing 100850, China

**Keywords:** tau protein, neurodegenerative diseases, post-translational modification, phosphorylation, therapeutic drugs

## Abstract

Tau protein is a microtubule-associated protein that is widely distributed in the central nervous system and maintains and regulates neuronal morphology and function. Tau protein aggregates abnormally and forms neurofibrillary tangles in neurodegenerative diseases, disrupting the structure and function of neurons and leading to neuronal death, which triggers the initiation and progression of neurological disorders. The aggregation of tau protein in neurodegenerative diseases is associated with post-translational modifications, which may affect the hydrophilicity, spatial conformation, and stability of tau protein, promoting tau protein aggregation and the formation of neurofibrillary tangles. Therefore, studying the role of tau protein in neurodegenerative diseases and the mechanism of aberrant aggregation is important for understanding the mechanism of neurodegenerative diseases and finding therapeutic approaches. This review describes the possible mechanisms by which tau protein promotes neurodegenerative diseases, the post-translational modifications of tau protein and associated influencing factors, and the current status of drug discovery and development related to tau protein, which may contribute to the development of new therapeutic approaches to alleviate or treat neurodegenerative diseases.

## 1. Introduction

In 1975, neuropathologists Kirschner MW et al. discovered a new protein in the brain tissue of patients with neurodegenerative diseases such as Alzheimer’s disease (AD) and Pick’s disease [1,2]. Tau protein is a microtubule-associated protein that is widely distributed in the central nervous system and maintains and regulates neuronal morphology and function [3]. Tau protein is composed of multiple interconnected repetitive units, which are each approximately 31–32 amino acid residues long and are connected to each other by short chains [4]. The primary sequence of tau consists of four functional regions: the N-terminal region, a proline-rich domain, microtubule-binding repeats (MTRs), and the C-terminal projection domain. There are six isoforms of tau in the mature human brain, including those with four microtubule-binding repeats (4Rs) with no amino-terminal inserts (0N), 4R1N, 4R2N, 3R0N, 3R1N, and 3R2N, which are derived from alternative splicing at exons 2, 3, and 10 of the pre-mRNA. In the healthy normal adult brain, all six isoforms are present with approximately equimolar 4R and 3R isoforms, whereas in the human foetal brain, only 0N3R tau is expressed [4,5,6,7].

An individual microtubule in the axon has a stable domain and a labile domain. Tau, a protein that has long been considered to be associated with the axon, binds to axonal microtubules and participates in the stabilisation of microtubules and axonal transport [8]. It is widely believed that tau stabilises microtubules in the axon [9,10,11,12], and that disease-induced loss of tau from axonal microtubules leads to their destabilisation [13,14,15]. Studies have found that tau is more abundant on the labile domain. The actual role of tau in regulating microtubule stability in the axon is not to stabilise axonal microtubules, but rather to allow them to have long labile domains [16,17]. Longstanding dogma is challenged by this new perspective on tau.

The abnormal aggregation and accumulation of tau protein is a pathological feature of a variety of neurodegenerative diseases, such as AD, frontotemporal dementia (FTD), and Parkinson’s disease (PD). The post-translational modification of tau protein also plays an important role in neurodegenerative changes. Research on the structure and function of tau protein and the mechanism by which it aggregates abnormally is of great significance for the treatment of related diseases; therefore, this review focuses on the roles of tau protein in neurodegenerative diseases, the post-translational modification of tau protein, the factors affecting the aberrant phosphorylation of tau protein, and advances in the research and development of drugs that use tau protein as a target.

## 2. Tau Protein and Neurodegenerative Diseases

Under physiological conditions, tau protein is soluble. Under nonpathological conditions, highly phosphorylated tau protein can be found in the foetal brain, in hibernating animals, and during anaesthesia-induced cryotherapy. However, when tau protein aggregates in a highly phosphorylated and insoluble form, it is considered pathognomonic for tauopathies. Pathological tau protein is in a highly phosphorylated state and is cleaved abnormally, resulting in an abnormal conformation and aggregation, which leads to neurofibrillary tauopathies [18,19,20]. The hyperphosphorylation of tau protein leads to its detachment from microtubules, and conformational changes lead to the formation of abnormal paired helices and straight filaments of tau protein, followed by the formation of tau protein aggregates. Different conformations of tau protein in the human body can cause tau protein lesions with different symptoms, and these different conformations resemble “prion strains”. Populations of tau conformers drive prion-like strain effects in Alzheimer’s disease and related dementias [21,22,23].

The pathological tau induces synaptic dysfunction in several ways, including reducing the mobility and release of presynaptic vesicles, decreasing glutamatergic receptors, impairing the maturation of dendritic spines at postsynaptic terminals, disrupting mitochondrial transport and function in synapses, and promoting the phagocytosis of synapses by microglia [24,25]. Additionally, tau has been reported to disturb autophagy function [26]. The disruption of the autophagic flux and the failure of autophagosomes to fuse with lysosomes result in the accumulation of vesicles, which can be observed in dystrophic neurites and cell bodies in tauopathy models [27,28,29].

Tau protein interacts with a variety of proteins, including amyloid-β (Aβ) and α-synuclein (α-Syn). The pathological diagnosis of tauopathy relies on the presence of particular tau inclusions with cell/brain-region-specific distributions [30]. PSP and CBD are characterised by inclusion bodies containing 4R tau protein, with clustered astrocyte and oligodendrocyte tangles in PSP and astrocyte plaques in CBD. PiD is characterised by filamentous 3R tau protein, known as Pick bodies, in neurons, occasional tau-positive branching astrocytes, and rare globular inclusion bodies in oligodendrocytes [31].

Neurodegenerative diseases involve increasing neuronal damage to the brain and spinal cord over time, characterised by a significant loss of specific neurons, with a progressive and ultimately fatal course. Neurodegenerative diseases are generally classified into two groups according to their phenotype: those that primarily affect the brain’s ability to learn and remember, such as AD, and those that primarily affect motor function, such as amyotrophic lateral sclerosis and cerebellar ataxia. Neurodegenerative diseases can also be divided into acute neurodegenerative diseases and chronic neurodegenerative diseases according to the duration of onset; the former mainly includes stroke and traumatic brain injury (TBI), and the latter mainly includes amyotrophic lateral sclerosis (ALS), Huntington’s disease (HD), PD, and AD. The dysfunction of tau protein in neurodegenerative diseases and neurotoxicity has received increasing attention in long-term studies of neurodegenerative diseases, and the regulation of tau protein is of great importance for maintaining normal neuronal function and combating neurodegenerative diseases.

### 2.1. Alzheimer’s Disease (AD)

AD is the most common neurodegenerative disease and has become a growing global health problem with tremendous impacts on individuals and society. According to the World Health Organization, approximately 50 million people worldwide suffer from AD, with approximately 10 million new cases each year. This number is predicted to increase to 152 million by 2050 as the population ages [32]. The two main pathological features of AD are extracellular β-amyloid (Aβ) deposition and intracellular hyperphosphorylation of tau protein, leading to the formation of aggregates and neurofibrillary tangles (NFTs). The number of NFTs is positively correlated with the degree of dementia in AD, and the paired helical filaments that are the major component of NFTs are formed by hyperphosphorylated tau protein [33]. Tau protein, whose abundance in the cerebrospinal fluid (CSF) increases more than 30 years before the onset of AD, is an integral part of the pathogenesis of AD.

In AD, the density of hyperphosphorylated tau tangles is closely associated with β-amyloid. β-amyloid induces the hyperphosphorylation of tau in neurons and causes neurofibrillary deposition [34], and β-amyloid oligomers can cause tau protein hyperphosphorylation, leading to cognitive decline [35].

Insulin signalling and tau hyperphosphorylation are important factors in the vicious cycle associated with cognitive decline in AD. Tau proteins are involved in insulin signalling, and insulin resistance also induces tau hyperphosphorylation and lesion formation. Under physiological conditions, insulin promotes the inhibition of serine phosphorylation through the PI3K-Akt signalling pathway via GSK3β, an important kinase involved in the insulin/PI3K/protein kinase B (insulin/PI3K/Akt) signalling pathway. Impaired insulin signalling can dysregulate the inhibition of GSK3β, which leads to tau hyperphosphorylation. Dysfunction of the insulin/PI3K/Akt signalling pathway, which regulates glucose metabolism, in the brains of AD patients can lead to the hyperphosphorylation of tau protein. High glucose regulates tau hyperphosphorylation and neuronal apoptosis through the TLR9-P38MAPK signalling pathway [36], and the hyperphosphorylation of tau protein and sustained hyperactivation of mTOR signalling in hyperglycaemia may be associated with the inhibition of caveolin-1 (Cav-1) [37].

In AD, tau protein was found to be glycosylated and O-GlcNAcylated before becoming aberrantly phosphorylated and N-linked-glycosylated at the N410 site of the tau 2N4R heterodimer. This aberrant N-linked glycosylation may have potential as a biomarker of early AD as well as a marker of AD progression [38].

High levels of tau protein with acetylation at the Lys163, Lys174, and Lys180 sites, which inhibits the degradation of hyperphosphorylated tau protein and thus contributes to the accumulation of phosphorylated tau protein, are detected in the brains of patients with mild to moderate AD. In addition, acetylation at the Lys274, Lys280, Lys281, and Lys369 sites was shown to impair tau function and to be increased in AD brains [39].

In AD, microglial proliferation is associated with tau protein pathology. In microglia, nucleotide-binding oligomerisation domain-, leucine-rich repeat-, and pyrin domain-containing protein 3 (NLRP3) inflammatory vesicles have been shown to actively regulate tau protein pathology and affect tau protein aggregation by regulating the intracellular and extracellular concentrations of different forms of tau protein to influence tau-protein-associated neurodegeneration [40]. *Apolipoprotein E (ApoE)* also plays a key role in the microglia-dependent and astrocyte-dependent regulation of tau pathology and tau-induced neurodegeneration [41,42].

White matter lesions (WMLs) are caused by a chronic insufficiency of cerebral perfusion; they are common in elderly individuals, closely related to cognitive decline, often found in the brains of patients with AD, and associated with ischaemia, hypertension, diabetes mellitus, and atherosclerosis in middle-aged and older adults. WMLs are associated with and coexist with the hyperphosphorylation of tau protein in the CSF of patients with AD [43,44]. WMLs promote the formation of NFTs [45]. In addition, postoperative cognitive dysfunction (POCD) is a serious complication of anaesthesia and surgery. Interestingly, WML is an important risk factor for POCD in humans, and tau protein hyperphosphorylation can be induced by surgery performed under general anaesthesia [46].

### 2.2. Parkinson’s Disease (PD)

PD is the second most common neurodegenerative disease, and the pathology of PD is characterised by the progressive loss of nigrostriatal dopaminergic neurons and the formation of neuronal Lewy bodies (LBs). LBs are mainly composed of aggregated α-synuclein (α-Syn). Tau and α-syn are colocalised in cellular axons, and in neuronal cells, they interact directly via binding sites in the c-terminal region of α-syn (55–140 aa) and the microtubule-binding region of tau (192–383 aa); as a result, α-syn stimulates protein kinase a to catalyse the phosphorylation of tau serine residues 262 and 356 [47]. This binding leads to an increase in the abundance of insoluble, high-molecular-weight α-syn and the co-localisation of aggregates of tau and α-syn. Increasing evidence suggests that tau proteins are also involved in the pathophysiological processes of PD. Lina Pan et al. demonstrated that tau proteins interact with α-Syn and accelerate its aggregation; that tau-modified α-Syn protofibrils have a stronger spreading activity than pure α-Syn protofibrils and induce mitochondrial dysfunction, synaptic dysfunction, and neurotoxicity; and that tau protein promotes the aggregation and spread of α-Syn in PD [48]. Autopsies of PD patients revealed that tau and α-Syn coexist in LBs and that tau-α-Syn interactions influence α-Syn pathology in PD [49].

### 2.3. Huntington’s Disease (HD)

HD is an autosomal dominant neurodegenerative disorder caused by polyglutamine expansion in the amino-terminal region of Huntington’s protein (Htt). Evidence of multiple changes in tau protein in the brain tissue of HD patients has been mounting in recent years. The reported tau protein alterations include an increase in total levels, an imbalance of isoforms produced by selective splicing (an increased 4R-/3R-tau ratio), and post-translational modifications (e.g., hyperphosphorylation or truncation) [50]. Mutant Htt and tau protein deposits occur in the same neuronal subpopulation, but the two proteins do not interact. It is not clear how mutations in the Htt protein-encoding gene lead to tau protein dysregulation in HD, and existing studies suggest that tau kinase and phosphatase play important roles. In HD patients and the R6/2 mouse model, the phosphorylation level of tyrosine 216 is increased; the expression of CaMKII, ERK1/2, and CDK5 is downregulated; and the activity of GSK-3β is reduced [51]. A reduced expression of calmodulin neurophosphatase has been observed in HD patients, and mutant Htt proteins can induce aberrant tau protein hyperphosphorylation in vivo via the dysregulation of calmodulin neurophosphatase [52]. A reduced expression of calmodulin phosphatase may be the cause of tau protein hyperphosphorylation in HD. There is substantial evidence for the presence of pathologic tau protein in HD, but further studies are needed to confirm the relevance of tau protein to HD and the efficacy of targeting tau protein as a therapeutic approach for HD.

## 3. Post-Translational Modifications of Tau Protein

Post-translational modifications of tau protein are strongly associated with neurodegenerative diseases. These post-translational modifications include phosphorylation, acetylation, glycosylation, nitrosylation, ubiquitination, heterodimerisation, and truncation [53,54] and can affect the structure and function of tau protein and thus its localisation and regulation in neuronal axons. Tau, a protein involved in several neurodegenerative diseases, has an intrinsic propensity to undergo liquid–liquid phase separation (LLPS) [55,56,57]. The main driving force for both homotypic and heterotypic LLPSs of tau appears to be electrostatic interactions that can be modulated by post-translational modifications (PTMs). Phosphorylation and acetylation have been demonstrated to exert a regulatory effect on LLPS [58,59,60,61]. In neurodegenerative diseases, aberrant post-translational modifications of tau protein result in the loss of normal regulatory functions, which negatively impact the normal functioning of neurons and contribute to the onset and progression of neurological disorders. The following section primarily focuses on the clinical development and more extensively studied modifications, namely phosphorylation, acetylation, methylation, and glycosylation.

### 3.1. Phosphorylation of Tau Protein

Tau protein structure and function are regulated through a variety of post-translational modifications, one of the most important of which is phosphorylation. Tau has a significant potential for combinatorial phosphorylation. The most common form of phospho-epitope in tau is phosphorylation at serine (S) or threonine (T) residues followed by proline (P). This occurs particularly within the proline-rich region (PRR) and C-terminal region (CTR) of tau, and includes 17 distinct SP or TP phosphorylation sites. Many of these sites are prominently phosphorylated in aggregated tau in neurofibrillary tangles [62,63,64,65,66]. Under normal conditions, protein kinases and protein phosphatases maintain tau protein at physiological levels. Based on the sequence characteristics of the protein kinases that catalyse the phosphorylation of tau protein, they can be classified as proline-dependent protein kinases (PDPKs) or nonproline-dependent protein kinases (non-PDPKs). PDPK can be roughly divided into three categories: ① mitogen-activated protein kinase (MAPK), such as extracellular signal-regulated kinase (ERK), c-Jun N-terminal kinase (c-Jun N-terminal kinase) (JNK), and p38 mitogen-activated protein kinases (p38MAPKs); ② cyclin-dependent kinase (CDK), such as CDK5; ③ and glycogen synthase kinase-3 (GSK-3). PDPK can be roughly divided into three categories: ①mitogen-activated protein kinase (MAPK) such as extracellular signal-regulated kinase (ERK), c-Jun N-terminal kinase (c-Jun N-terminal kinase) (JNK), and p38 mitogen-activated protein kinases (p38MAPK); ② cyclin-dependent kinase (CDK), such as CDK5; and ③ glycogen synthase kinase-3 (GSK-3). Non-PDPKs include calcium/calmodulin-dependent protein kinase II (CaMK II), protein kinase A (PKA), dual-specificity tyrosine-phosphorylation regulated kinase 1A (DYRK1A), adenosine monophosphate-activated protein kinase (AMPK), microtubule-affinity regulating kinase (MARK), protein kinase C, Ser/Thr protein kinase B, tyrosine kinase 1, and tyrosine kinase 2 [67].

Tau protein has more than 80 phosphorylation sites, and 4 of these sites (T50, T69, T181, T205) and two protein kinases (GSK3β, p38a) are closely related to the regulation of tau protein phosphorylation. These four sites are known as “master sites”, with T50, T69, and T181 exerting the most extensive regulatory effects on the other sites. There is an interdependent relationship among tau phosphorylation sites, with the phosphorylation of an initial site affecting the phosphorylation of subsequent sites. In this pattern of “dependent regulation”, the phosphorylation of the key sites T50, T69, and T181 can regulate the phosphorylation of other sites, thus regulating the overall phosphorylation level of tau protein. Tau kinases have two modes of operation: the direct mode, in which the kinase interacts directly with tau protein, and the indirect mode, in which tau phosphorylation needs to be mediated by other factors. p38a and Cdk5 act directly on specific sites, while GSK3β does not bind directly to the phosphorylation sites and instead functions via the indirect mode [36].

Protein phosphatases include protein phosphatase-1 (PP1), protein phosphatase 2A (PP2A), and protein phosphatase 2B (PP2B). PP2A is a major phosphorylated serine/threonine phosphatase that is a key regulator of cellular signalling pathways. PP2A regulates a variety of signalling pathways, including the cell cycle, cell growth and development, and cytoskeletal dynamics. Tau protein dephosphorylation is enhanced when PP2A activity is increased. PP2A deficiency in AD is tightly correlated with tau protein hyperphosphorylation [68]. GSK-3β and PP2A can regulate each other and control tau protein phosphorylation directly or indirectly. GSK-3β can inhibit PP2A by increasing the inhibitory phosphorylation of Tyr307 and decreasing the expression of PP2A, and the interactions between the PI3K-AKT-GSK-3β and PP2A pathways should not be ignored. The former regulates the methylation of PP2A through GSK-3β [69]. The upregulation of GSK-3β leads to an increased methylation and activity of PP2A; PP2A also regulates GSK-3β phosphorylation, and the downregulation of PP2A enhances the Ser9 phosphorylation of GSK-3β and inhibits its kinase activity [70]. In addition, the Src family plays an important role in tau protein hyperphosphorylation [71], as PP2A can be regulated via tyrosine phosphorylation mediated by Src and Fyn kinase (an Src family kinase) [72,73].

However, when the regulation of protein kinases and protein phosphatases is altered, tau protein and microtubules depolymerise, and hyperphosphorylated tau protein accumulates abnormally. The accumulation of phosphorylated tau protein leads to synaptic damage, neuronal dysfunction, and the formation of NFTs. However, how the overall phosphorylation level of tau protein can be regulated by balancing the phosphorylation levels of these sites and how the phosphorylation of tau protein becomes imbalanced and triggers disease remain to be explored.

### 3.2. Acetylation of Tau Protein

Acetylation represents a pivotal post-translational modification (PTM) that exerts regulatory influence over both histone and non-histone proteins. It is implicated in the control of a multitude of cellular processes, including DNA transcription, RNA modifications, autophagy, proteostasis, aging, the regulation of cytoskeletal structures, and metabolism. Specific acetylation sites regulate the formation of tau aggregates, synaptic signalling, and long-term potentiation [74,75]. The decrease in the acetylated form of tau is neuroprotective [76]. Acetylation has been demonstrated to strongly attenuate the aggregation of four-repeat tau protein, while promoting amyloid formation of three-repeat tau. Furthermore, the acetylation of lysine 298 has been identified as a hotspot for isoform-specific tau aggregation [77]. Acetylation is essential for maintaining neuronal plasticity and is therefore critical for learning and memory. The homeostasis of acetylation is maintained via the activities of histone acetyltransferase (HAT) and histone deacetylase (HDAC) enzymes, and both hyper- and hypoacetylation can disrupt neurophysiological homeostasis. In some neurodegenerative diseases, including AD, PD, HD, and ALS, acetylation increases the accumulation of related pathophysiological proteins such as tau, α-synuclein, and Htt. In addition, the dysregulation of acetylation is associated with impaired axonal transport, a key pathological mechanism in ALS [78]. 

The acetylation of tau has become known as a key pathogenic modification; in particular, acetylation of the K280 site of the hexapeptide 275VQIINK280, a key sequence, drives tau aggregation. Tau protein acetylation can impact phosphorylation via different residues, and acetylation at the K280 site due to pathological acetyltransferase activity is a determinant of tau phosphorylation at certain residues. However, the complex mechanism that underlies the relationship between tau acetylation and tau phosphorylation is currently unknown [79].

Tau protein also has intrinsic acetyltransferase activity that mediates self-acetylation. Tau protein can be acetylated by histone acetyltransferase p300 (EP300) or CREB-binding protein (CBP) and deacetylated by sirtuin 1 (SIRT1) and histone deacetylase (HDAC6) at microtubule-binding repeats. More than 20 Lys residues within the sequence can be deacetylated [80]. Increased tau acetylation at the K274 and K281 loci has been observed in the brain in AD patients and animal models, and acetylation at the K274/281 locus is an important site of post-translational modification for tau neurotoxicity [81]. 

### 3.3. Glycosylation of Tau Protein

N-glycosylation is defined as the addition of an N-acetylglucosamine (GlcNAc) to the peptide backbone via a β-1N linkage to the nitrogen atom of an asparagine (Asn) [82]. The initial process occurs within the endoplasmic reticulum (ER), where the N-glycan precursor is synthesised from glucosamine (GlcNAc) and mannose. Consequently, mannose and glucose units are incorporated to form a glycan structure comprising 14 sugars. Thereafter, the sugar chain is transferred to a protein bearing the sequence Asn-X-serine (Ser)/threonine (Thr). Subsequently, the added glycan is trimmed by mannosidases and glucosidases in the endoplasmic reticulum (ER), and further modified by glycosyltransferases in the Golgi apparatus to ensure glycan maturity [83,84]. The abnormal deposition of tau protein is an important pathological feature of neurodegenerative diseases, and the degree of tau glycosylation is also an important research focus. Studies have shown that glycosylation modifications can increase tau protein aggregation, promote abnormal tau protein deposition, and further activate neuronal apoptosis signalling pathways. Glycosylation is an enzymatic process that involves the covalent attachment of glycans to the side chains of proteins. The three types of glycosylation, N-linked glycosylation, O-linked glycosylation, and O-GlcNAcylation, play important roles in neurodevelopment and neurodegenerative diseases. N-linked glycosylation involves the attachment of N-linked glycans (N-acetylglucosamine/GlcNAc) to glycoproteins at an Asn-X-Ser/Thr site via the nitrogen atom of the Asn residue. Based on the extensions present on the N-linked glycans, they can be classified into three groups, namely, high-mannose, complex, and heterodimeric N-linked glycans [85]. High-mannose N-linked glycans consist of a core with additional Man residues, complex N-linked glycans are characterised by a core with additional GlcNAc residues, and heterozygous N-linked glycans have a core with additional Man and GlcNAc residues. This process is important for the normal function and localisation of proteins, and N-linked glycosylated tau proteins are present in the brain tissue in AD but not in healthy brain tissue [86].

### 3.4. Methylation of Tau Protein

Methylation is carried out by the class of enzymes known as methyltransferases. Class II methyltransferases are SET-domain-containing enzymes that primarily function as histone methyltransferases. However, there are methyltransferases of the same class, such as G9a and SUV39, which shuttle between the nucleus and cytoplasm to act on cytoplasmic proteins [87,88]. There is a direct relationship between methylation and other lysine modifications, particularly acetylation and ubiquitination, in tau protein. The occupancy of a single lysine residue with methylation, acetylation, or ubiquitination may influence the fate of tau protein in different ways [89]. Methylation is an important post-translational modification of tau protein. The post-translational modification state determines the function and stability of the tau protein, and the lysine methylation profile of tau is altered once it adopts a pathologically aggregated form. However, tau methylation can compete with other post-translational modifications, such as acetylation and ubiquitination. On the one hand, tau protein methylation is protective, and both the acetylation and methylation of lysine residues are important for tau protein function and stability. Tau protein methylation can be protective because, while multiple sites of tau are heavily acetylated in the AD brain, these sites are preferentially methylated [90]; enzymes involved in this protective function, such as PP2A, can be regulated by methylation, and the inhibition of PP2A leads to increased and aberrant levels of phosphorylation in combination with reduced intracellular methylation, resulting in the hyperphosphorylation of tau protein. On the other hand, tau protein methylation may have deleterious effects. Tau methylation regulates microtubule binding and enhances prion-like tau protein aggregation. Filamentous tau protein aggregates isolated from AD patients are methylated on multiple lysine residues, but the exact methyltransferase is not yet known [91].

## 4. Factors Influencing the Hyperphosphorylation of Tau Protein

**Figure 1:**
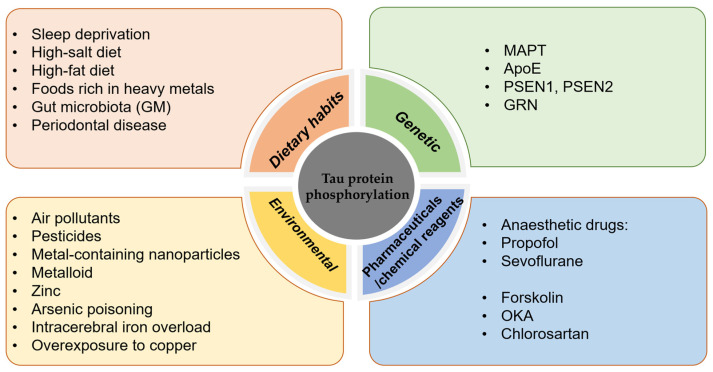
Factors influencing the hyperphosphorylation of tau protein.

The phosphorylation of tau protein plays an important role in post-translational modifications, and in-depth studies on the factors affecting tau protein phosphorylation can help to better elucidate the mechanisms of neurodegenerative diseases and provide new ideas for the development of related therapeutic approaches(see Figure 1 below). 

### 4.1. Dietary Habits

Poor lifestyle and dietary habits are strongly associated with the development of tauopathies. Sleep deprivation, which increases the risk of developing tauopathies, increases the concentration of tau in the CSF, and the effect on tau protein phosphorylation varies depending on the modification site [92,93].

High salt intake promotes tau protein phosphorylation in the mouse brain [94], and a high-salt diet leads to a decrease in NO levels, which directly leads to an increase in the activity of calcium-activating enzymes (calpains), which cleave p35 to p25, resulting in the abnormal activation of CDK5 and excessive phosphorylation of tau protein [95,96], which in turn leads to cognitive impairment. A high-fat diet activates neuron-specific transcription factors (C/EBPβ) and their downstream products (AEP enzymes)—collectively known as the C/EBPβ/AEP neural signalling pathway—leading to the aggregation of hyperphosphorylated tau protein and cognitive impairment [97]. A high-fat diet also activates serum/glucocorticoid-regulated kinase 1 (SGK1) and GSK-3β through phosphorylation of the Ser214 site of tau protein, enabling the formation of the SGK1-GSK-3β-tau complex, which contributes to pathological changes in tau protein [98]. A diet low in saturated fat may be beneficial for the prevention of Parkinson’s disease, according to findings from the Swedish National March Cohort [99]. Foods rich in heavy metals, such as lead, zinc, aluminium, and mercury, poison brain cells, and the higher the exposure is, the higher the incidence of mental decline and dementia. In addition, zinc induces PP2A inactivation and tau protein hyperphosphorylation via an Src-dependent pathway. Animal studies and epidemiological investigations have found that contaminants in the diet can cause neurotoxic effects [100,101,102].

The gut microbiota (GM), the most densely populated bacterial community colonising the human body, is involved in the pathology of AD [103]. Outer membrane vesicles (OMVs) are nanosized vesicles produced by the GM. OMVs increase the permeability of the blood-brain barrier and promote the activation of astrocytes and microglia, which in turn induces an inflammatory response and the hyperphosphorylation of tau protein via activation of the GSK-3β pathway, ultimately leading to cognitive impairment [104]. David Holtzman’s group found that gut microbes promote tau-protein-mediated neurodegeneration in an *ApoE* genotype-dependent manner and that short-chain fatty acids secreted by gut microbes are involved in the regulation of tau pathology by the gut flora [105]. Adherence to the Mediterranean-DASH Intervention for Neurodegenerative Delay (MIND) dietary pattern has been associated with a reduced risk of cognitive impairment, as demonstrated by epidemiological studies [106,107,108].

Poor oral hygiene predisposes patients to periodontal disease, and periodontitis is an important factor that increases the risk of developing AD [109,110]. Porphyromonas gingivalis (PG) is the main causative agent of periodontitis, and PG infection leads to tau hyperphosphorylation [111], with increased phosphorylation at the Ser396 [112] and Thr231 sites, which plays an important role in NFT lesions in AD [113]. Tau protein is a substrate for gingival peptides secreted by PG [114]. PG OMVs increase vascular permeability and trigger the formation of NLRP3 inflammatory vesicles [115], and PG crosses the blood-brain barrier to induce neuroinflammation. Tau protein phosphorylation, memory dysfunction, and OMV are important factors during the induction of AD pathology by PG [116]. Olsen et al. found that lipopolysaccharide (LPS) from PG activated the PI3K/AKT pathway, increased glycogen synthase kinase-3β (GSK-3β) mRNA expression, and inhibited GSK3β [117], and the inhibition of GSK3β activation may delay the pathologic progression of periodontitis-induced AD [118]. Clostridium nucleatum-induced periodontitis can also lead to the exacerbation of AD symptoms, including increased cognitive impairment, β-amyloid accumulation, and tau protein phosphorylation in the mouse brain [119]. The results of an observational cohort study indicated that periodontitis is associated with an increase in cognitive decline in Alzheimer’s disease [120]. The relationship between periodontitis and Alzheimer’s disease is bidirectional [121]. Therefore, one effective way to prevent AD is to maintain a good diet and good oral hygiene.

### 4.2. Genetic Factors

Tau protein hyperphosphorylation is an important pathological process in neurodegenerative diseases involving multiple genes.

#### 4.2.1. *MAPT*

The *MAPT* gene encodes tau protein, and variants of this gene have been associated with the abnormal aggregation of tau protein and the development of neurodegenerative diseases. The *MAPT* gene is located on the long arm of human chromosome 17 and contains 16 exons and multiple introns [122]. More than 40 *MAPT* gene variants are known, including variants with mutations, insertions, deletions, etc. The abnormal aggregation of tau protein has been associated with a variety of neurodegenerative diseases, such as AD, FTD, and corticobasal ganglionic degeneration, and mutations in the *MAPT* gene are thought to be an important cause; thus, the *MAPT* gene has significance for the study of neurodegenerative diseases [123]. A meta-analysis of genome-wide association studies identified ancestry-specific associations underlying circulating total tau levels, indicating an important role of *MAPT* in many neurodegenerative diseases. The analysis confirmed the strong association in Europeans of the 17q21 *MAPT* locus (lead genetic variant rs242557) [124]. Mutations in the *MAPT* gene are found in FTD and frontotemporal degeneration with parkinsonism linked to chromosome 17 (FTDP-17). Such mutations or abnormalities in the *MAPT* gene can lead to aberrant phosphorylation, aggregation, and the deposition of tau protein, which can in turn impair neuronal cell functions [125].

#### 4.2.2. *ApoE*

The apolipoprotein E (*ApoE*) gene is an important risk factor for and regulator of the development of AD [126,127,128]. Increasing age and the *ApoEε4* genotype cause women to be prone to late-onset AD [129,130]. Women have a higher risk of dementia than men, and they also have high tau levels than men; in addition, female carriers of *ApoEε4* with some degree of cognitive impairment have higher levels of both total tau and phospho-tau (p-tau) in the CSF [131]. It has been found that the oestrogen receptor (oestrogen receptor, ESR) has an important influence on the development of AD in women with the *ApoEε4* genotype and that the oestrogen receptor and *ApoEε4* interact with each other [132,133,134]. The oestrogen receptor increases the expression of nerve growth factor and its receptor and inhibits the abnormal phosphorylation of tau protein, thus reducing the risk of AD in individuals. When ovarian failure occurs and oestrogen, which is a protective factor, is absent, women are at increased risk of developing cognitive impairment. Moreover, oestrogen deficiency mediates an increase in PP2A phosphorylation (Y307), which disrupts the dephosphorylation of abnormally hyperphosphorylated tau protein, leading to NFT formation [135]. X-linked ubiquitin is also known to increase the phosphorylation of PP2A. Moreover, X-linked ubiquitin-specific peptidase 11 (USP11) has been shown to increase susceptibility to tauopathy in women. By using in vitro and in vivo models and human AD brain tissue, Yan Y et al. demonstrated that USP11 initiates tau protein deubiquitination via lysine-281. Enhanced pathological tau protein aggregation and deubiquitination activate a pathway for tau protein acetylation on lysines 281 and 274. In a mouse model of tauopathy, elimination of the USP11 gene preferentially protects females against acetylated tau accumulation, tau pathology, and cognitive impairment. USP11 levels in females are also strongly associated with tau pathology, and the inhibition of USP11-mediated deubiquitination of tau protein may provide an effective therapeutic approach to protect females from increased susceptibility to AD and other tauopathies [136]. Emerging research has shown racial and ethnic variations in the magnitude of association between the apolipoprotein *ApoEε4* allele and the risk of developing Alzheimer’s disease and related dementias (ADRDs). The frequency of *ApoEε4* carriers was found to be highest among the African American population, followed by the African population, the white population, the Hispanic/Latino population, and the Chinese population [137].

#### 4.2.3. Others

In addition to the *MAPT* and *ApoE* genes, numerous genetic variants have been associated with the abnormal aggregation of tau protein and the development of neurodegenerative diseases. For example, gene mutations in the premature ageing proteins *PSEN1, PSEN2* [138,139], and GRN [140,141] have been shown to be associated with abnormal tau protein phosphorylation, which promotes the abnormal aggregation of tau protein, ultimately leading to the onset and development of neurodegenerative diseases.

### 4.3. Environmental/Chemical Factors

Exposure to several environmental factors, such as air pollutants, pesticides, metal-containing nanoparticles, and many metalloids, such as arsenic, mercury, lead, and cadmium, may increase the risk of tau protein hyperphosphorylation.

Arsenic poisoning poses a significant threat to human health, and low concentrations of arsenic can impair neurologic function [142]. The primary route of human exposure to arsenic is through drinking water; the permissible concentration of arsenic in drinking water is 10 μg/L, and it is estimated that 100 million people worldwide are exposed to excessive amounts of arsenic through drinking water. Arsenic is involved in cellular signalling cascades involved in regulating tau protein function or hyperphosphorylation, which ultimately leads to neurological damage [143]. Zinc is a heavy metal ion that is widely distributed in the normal brain and accumulates in susceptible regions in the AD brain. Zinc induces PP2A inactivation and tau protein hyperphosphorylation via an Src-dependent pathway [144] and can mediate CDK5-Tyr15 phosphorylation to induce the activation of CDK5 [145]. In AD, intracerebral iron overload is a key pathological feature that occurs in conjunction with tau hyperphosphorylation, and iron is involved in the hyperphosphorylation of tau protein through dysfunctional insulin signalling. Ferrous chloride (Fe^2+^) leads to the aberrant phosphorylation of tau and decreases tyrosine phosphorylation levels of insulin receptor β (IRβ) and insulin signalling substrate 1 (IRS-1) via the phosphatidylinositol PI3K kinase p85a subunit. The hyperphosphorylation of tau and disruption of insulin signalling in the brain were reported to be induced in iron-overloaded mice [146]. Overexposure to copper is thought to be associated with the development of AD neuropathology, and increased tau phosphorylation with the aberrant activation of cdk5/p25 plays an important role in the development of copper-induced tau pathology [144]. Elevated intracellular calcium levels also promote the hyperphosphorylation of tau protein, ultimately leading to NFT formation [147]. The role of metals in the development of neurodegenerative diseases has been demonstrated by a substantial body of research. Epidemiological and experimental studies have demonstrated that exposure to heavy-metal pollutants has a long-term effect on the central nervous system (CNS), potentially through epigenetic modifications, which can stimulate the development of neurodevelopmental disorders and neurodegenerative diseases [148,149,150].

Anaesthetic drugs such as propofol and sevoflurane can cause an abnormal phosphorylation of tau protein in the hippocampus and are closely associated with degenerative changes in the central nervous system. Propofol-mediated cognitive dysfunction in rats may be related to the hyperphosphorylation of tau protein, leading to the re-entry of neuronal cells into the cell cycle, which results in apoptosis [151]. In addition, sevoflurane anaesthesia-induced postoperative cognitive deficits are associated with the hyperphosphorylation of tau [152]. Sevoflurane treatment induces neuroinflammation by upregulating the expression of full-length *ApoE* and *ApoE*-containing 18 kDa fragments in naive hippocampal neurons and by increasing tau protein phosphorylation [153].

The PKA-specific activator forskolin induces the hyperphosphorylation of tau protein at the Ser214, Ser396, and Ser202/Thr205 sites in cultured primary hippocampal neurons [154]. Okadaic acid (OKA), a polyether C38 fatty acid toxin derived from the black sponge *Hallichondria okadaii*, is a potent and selective inhibitor of the PP1 and PP2A protein phosphatases, and OKA has proven to be a potent tool for the study of various regulatory mechanisms and neurotoxicity associated with the hyperphosphorylation of tau protein [155]. Chlorosartan-induced hypotension leads to tau protein hyperphosphorylation and memory impairment, possibly via oxidative stress-induced tau protein hyperphosphorylation and dendritic spine loss, increasing the risk of AD-like pathological changes and behavioural disorders.

## 5. Progress in the Development of Therapeutic Drugs Targeting Tau Protein

The inability to target Aβ in AD therapy and the intractability of neurodegenerative diseases have led many researchers to focus their research on tau protein, and many basic studies have demonstrated that the knockdown or inhibition of tau can reduce tau-associated pathological effects; thus, drugs targeting tau protein have attracted increasing attention. Numerous drugs are being developed and studied for the treatment of neurodegenerative diseases associated with the aberrant aggregation of tau protein, and the mechanisms of action of these drugs include the inhibition of aberrant post-translational modifications of tau protein, facilitation of the clearance and degradation of tau protein, and prevention of the aberrant aggregation of tau protein. Although no tau protein-targeted drug has been approved for the market, some progress has been made in related clinical trials (Table 1). The comprehensive review by Sigurdsson and colleagues provides an overview of clinical trials involving drugs that target tau. This section thus presents an overview of tau-targeted therapy, along with a discussion of potential new therapeutic avenues for consideration [156].

### 5.1. Drugs Targeting Post-Translational Modifications of Tau Protein

Targeting the phosphorylation of tau protein is a potential treatment approach for AD, and while many drugs have been tested in preclinical studies, few have entered clinical trials; these include lithium salts, tideglusib, saracatinib, nilotinib, and others. Lithium salts are commonly used for psychiatric disorders, and the results of three clinical trials of the use of lithium salts for the treatment of AD have been published, but lithium salts failed to demonstrate neuroprotective activity or improvements in cognitive or psychometric scores and did not affect CSF Aβ42, pTau181, pTau231, or total tau levels [174]. Tideglusib, a small-molecule GSK3β inhibitor, failed to improve cognition in a phase 2 clinical study, and CSF levels of Aβ42, total tau, and pTau181 were not altered, resulting in failure of the trial [175,176]. Saracatinib and nilotinib are small-molecule inhibitors of Fyn and Bcr-Abl, respectively. Saracatinib is a small-molecule inhibitor of Fyn kinase that reduces tau aggregation, increases synaptic density, and improves memory [177,178,179,180]. Saracatinib failed to demonstrate statistically significant efficacy in phase 1b and phase 2 clinical trials [178]. Nilotinib, a small-molecule inhibitor of Bcr-Abl, had some effect on CSF and frontal cortex amyloid levels in a phase 2 clinical trial but failed to exert an effect on tau [181,182,183]. Cornel iridoid glycoside (CIG) is a major component extracted from *Cornus officinalis*. CIG inhibits GSK-3β activity by activating the P13K/AKT and PP2A signalling pathways. In addition, CIG inhibited GSK-3β activity to enhance PP2A activity via suppression of the PME-1-induced demethylation of PP2Ac, thereby modulating the crosstalk between the GSK-3β and PP2A signalling pathways and thus inhibiting the hyperphosphorylation of tau protein. These results suggest that CIG may be a promising therapeutic agent for AD treatment [184]. Chronic low-dose sodium selenate reduced tau protein phosphorylation in cell culture and mouse disease models and is considered a promising lead compound for tau-targeted therapy. Sodium selenate reduces tau protein phosphorylation by activating PP2A, but the exact mechanism remains to be elucidated. However, sodium selenate (selenate) failed to produce improvements in cognition in a phase 2 clinical study. Due to insufficient clinical evidence, it cannot be assumed that enhancing phosphatase activity is beneficial in the treatment of AD [185,186]. Hao Wang’s team designed exosomes carrying curcumin (cur) to enable curcumin to cross the blood-brain barrier (BBB) and inhibit the phosphorylation of tau protein by activating the AKT/GSK-3β pathway, better preventing neuronal death in vivo and in vitro and alleviating AD symptoms [187]. By inhibiting activation of the JNK signalling pathway, isorhynchophylline (IRN) improves cognitive function by reducing Aβ production and deposition, tau protein hyperphosphorylation, and neuroinflammation and has potential as a pharmacological treatment aiming to prevent further progression to AD [188].

Targeting the acetylation of tau protein is also a potential means of treating AD. Acetylation at the K274/281 site is an important post-translational modification for tau neurotoxicity, and BGP-15 is a potential therapeutic agent for AD [81]. Tau acetylated on lysine 280 (tau-ack280) is essential for tau protein secretion, aggregation, and seeding, and researchers have found that the monoclonal antibody Y01 prevents the lysine 280-acetylated tau-protein-induced progression of tauopathy in cell and mouse models. The researchers developed an antibody, Y01, that specifically targets tau-acK280 and solved the crystal structure of the Y01-acK280 peptide complex; upon interaction with acetylated tau protein aggregates, Y01 prevented the progression of tauopathy and increased neuronal viability. The Y01 antibody that specifically recognises acK280 is a promising candidate for the treatment of AD and other neurodegenerative diseases associated with tauopathy [189].

The methylation of tau protein is a contributing factor in tau pathogenesis and may be a potential therapeutic drug target for the treatment of different tauopathies. One strategy to study the site-specific effects of methylation is to create methylation mimics that replace lysine (K) with a hydrophobic moiety (phenylalanine (F)) to approximate the effect of lysine methylation (C). A tau methyl mimic was used in this study to mimic several functional aspects of tau methylation, such as effects on microtubule binding and tau aggregation, in cellular models [91].

### 5.2. Tau Protein Aggregation Inhibitors

Methylene blue blocks the polymerisation of tau in vitro by trapping the tau monomers in an aggregation-incompetent conformation. The methylene blue derivative LMTX (TRx0237) is a small-molecule tau protein aggregation inhibitor that prevents tau protein aggregation or degrades preexisting aggregates [190]. The drug has failed to achieve positive results in the three clinical studies that have been completed to date, and there is still one phase 1/3 clinical study underway [191]. Curcumin is a natural product derived from the Curcuma longa plant and has been utilised in culinary practices and herbal medicine for centuries. Its potential for clinical development is evident, as it has been demonstrated to reduce tau and Aβ pathology and ameliorate cognitive deficits. Curcumin reduces tau lesions in animal models and prevents tau accumulation in vitro. A phase II clinical trial (NCT01383161) examined the effects of curcumin therapy on patients with MCI and healthy adults, and individuals who received the drug showed improvements in long-term memory, visual memory, and attention. In addition, a significant association was observed between improved cognitive ability and a reduced binding of PET ligands to pathological tau and Aβ. The phase 2 study (NCT01811381) is examining the effects of curcumin alone or in combination with exercise in patients with MCI or subjective cognitive impairment. The study endpoints included blood-based biomarkers, changes in PET imaging, and adverse events. This study was due to be completed in 2020, but the results have not yet been published [192,193]. Natural products, such as caffeine, CIG, and catechins [184], have been shown to inhibit the abnormal aggregation of tau protein and are being further investigated for their potential to treat neurodegenerative diseases. However, these drugs are still in the research and development stage and have not yet been approved for clinical use.

### 5.3. Others

In addition to the drugs discussed above, anti-tau protein monoclonal antibodies are a promising therapeutic strategy, but further exploration of their mechanism of action and side-effects is still needed [194]. Nanoliposomes and exosomes, as smart drug delivery systems capable of crossing the blood-brain barrier (BBB) and targeting brain tissue, have broad application prospects [195]. 1,6-O,O-diacetylbritannilactone (OABL), a 1,10-seco-eudesmane sesquiterpene lactone isolated from the herb *Inula britannica* L., exhibited strong anti-inflammatory activity in vitro as well as favourable BBB penetration properties. OABL significantly reduced the phosphorylation of tau protein [196]. In order to simulate the structure of natural nanoparticles, Yang Ding et al. grafted endogenous apolipoprotein A-I and its mimicking peptide 4F fused angiopp-2 (Ang) onto lipid nanocomposite (APLN) in sequence, and further assembled methylene blue (MB) into APLN (APLN/MB) to inhibit tau aggregation. APLN/MB “drug carrier” synergistic therapy is a promising approach for the development of Alzheimer’s disease treatment [197]. Romany Abskharon’s team designed a bispecific nanobody, consisting of a nanobody targeting a receptor on the BBB and a nanobody tau capping inhibitor linked by a flexible linker, that can improve blood–brain barrier penetration and blocked seeding by recombinant tau oligomers [198]. Joanna M Wasielewska’s team has found a new approach. Focused ultrasound applied together with microbubbles (FUS + MB) is a novel technique to transiently open the BBB and enhance drug delivery, developing the ultrasound-mediated delivery of aducanumab and anti-Tau antibodies [199]. Jingfen Su’s team have conceptualised a strategy, called dephosphorylation-targeting chimeras (DEPTACs), for specifically hijacking phosphatases to tau to debilitate its hyperphosphorylation [200].

AADvac1, a tau fragment peptide vaccine, was shown to produce antibodies in 95% of subjects in a phase 2 clinical study. AADvac1 significantly slowed the growth of NFTs in the blood, with a significant reduction in CSF pTau217 levels and a trend towards a reduction in pTau181 and total tau levels in the treatment group, but no cognitive benefit was observed [201]. Bepranemab is a humanised IgG4 monoclonal antibody that binds to the central region of the tau protein, and semorinemab is an IgG4 antibody that targets extracellular tau to limit inflammatory microglial activation. Both are in phase 2 clinical trials [202]. Some antiviral drugs, such as lamivudine and acyclovir, have also been shown to inhibit the aberrant aggregation of tau protein and need to be further investigated for their potential in treating neurodegenerative disorders. Tau protein antibody therapies use antibodies directed against tau protein to mediate the removal and degradation of abnormally aggregated tau protein. Vallitinib, an epidermal growth factor receptor (EGFR)/HER2 inhibitor, has therapeutic potential in the context of lipopolysaccharide- and hyper-repeat-protein-induced neuroinflammatory responses and the early stages of tau protein pathology. Jieun Kim et al. showed that vallitinib has therapeutic potential for treating neuroinflammatory responses and the early stages of tau protein pathology induced by LPS and tau protein. Vallitinib inhibits EGFR/HER-2 via DYRK1A and ameliorates the early stages of neuroinflammatory responses and tau protein pathology [203].

Gene-silencing-based therapeutic options are also attracting attention, and Catherine J. Mummer’s research team used MAPTRx, an antisense oligonucleotide (ASO) drug that degrades the mRNA of the *MAPT* gene (which encodes the tau protein). They found that patients with mild AD who were treated with high doses of MAPTRx had an average reduction in tau concentrations in the CSF of more than 50 percent 24 weeks after the last dose of the drug. This was the first clinical attempt to use a gene-silencing-based drug for a dementia-related disease such as AD [204].

Proteolysis-targeting chimeras (PROTACs) represent an emerging paradigm-shifting technology. Currently, PROTAC is a highly active area of drug development. In order to achieve a more accurate regulation of protein function, Crews laboratory developed another novel regulatory tool after PROTAC, namely Phosphorylation Targeting Chimeras (PhosTACs). The researchers successfully achieved the dephosphorylation of tau protein with PhosTAC and confirmed that this approach can reduce the pathogenic pathological properties of tau protein, laying a foundation for the future treatment of Alzheimer’s disease [205,206,207].

## 6. Outlook

The study of tau protein has long been an important research direction in the field of neurodegenerative diseases. Tau protein is an important pathological feature in neurodegenerative diseases, and it is very important to study its structure and function and its specific mechanism of action in neurodegenerative diseases and to explore the relationship between tau protein and other proteins related to neurodegenerative diseases. To date, the study of tau protein has mainly focused on the mechanism of tau protein aggregation, including tau protein phosphorylation and tau protein aggregation, and it remains to be explored how tau protein becomes imbalanced and causes disease.

The treatments for tau protein-related diseases mainly involve the inhibition of abnormal tau protein aggregation and the degradation of tau protein aggregates. Several studies have demonstrated the effectiveness of therapeutic strategies such as anti-tau protein immunotherapy, pharmacological interventions, and gene therapy, and these advances have provided new ideas and approaches for the treatment of tau protein-related diseases. However, there are still limitations in the research and development of therapeutic approaches targeting the tau protein, which is a highly complex protein with different splicing isoforms and post-translational modifications that can affect the structure and function of the tau protein. In addition, various neurodegenerative diseases can lead to the abnormal aggregation of tau protein and neuronal damage due to different genetic and environmental factors. The abnormal aggregation of tau protein and neuronal damage are key processes in the onset and progression of neurodegenerative diseases, and treatment during the therapeutic window is of great importance for the effectiveness of tau protein-targeted therapy. At the same time, it is necessary to support the development and application of tau protein-targeted therapeutic approaches and to solve the problems associated with tau protein therapy, including immune reactions, cytotoxicity, and other adverse effects. This will enable the development of safer and more effective drug treatment strategies so that related therapeutic drugs can be more widely studied and applied in the future.

## Figures and Tables

**Table 1:** Drugs currently undergoing clinical trials for the treatment of tau-targeted therapies.

Drug Type	Drug Name (Ongoing Clinical Trials)
**Reducing tau expression**	NIO752, BIIB080 [157]
**Phosphatase modifiers**	Memantine [158], Sodium selenate [159]
**Kinase inhibitors**	Tideglusib [160], Lithium [161]
**Tau aggregation inhibitors**	Curcumin [162]
**Active immunisation**	AADvac1 [163], ACI-35 [164]
**Passive immunisation**	APNmAb005 [165], Bepranemab [166], Lu AF87908 [167], E2814 [168], BIIB076 [169], BIIB092 [170], MK-2214 [171], PRX005 [172], JNJ-63733657 [173]
